# Multimodal treatment and tumour biology-driven long-term survival in PSC-associated hilar cholangiocarcinoma: A case report

**DOI:** 10.3892/mi.2026.295

**Published:** 2026-01-09

**Authors:** Evangelia Florou, Michael Heneghan, Debashis Sarker, Parthi Srinivasan, Andreas Prachalias

**Affiliations:** 1 Institute of Liver Studies, Department of Hepato-Pancreato-Biliary Surgery, King s College Hospital, London SE5 9RS, UK; 2 Institute of Liver Studies, Department of General Hepatology and Liver Transplantation, King s College Hospital London, London SE5 9RS, UK; 3 Institute of Liver Studies, Department of Oncology, King s College Hospital London, London SE5 9RS, UK; 4 Hepato-Pancreato-Biliary Surgery and Liver Transplantation, London Bridge Hospital, London SE1 2PR, UK

**Keywords:** primary sclerosing cholangitis, hilar cholangiocarcinoma, liver transplantation, tumour biology, multimodal treatment, long-term survival, metastatic recurrence

## Abstract

Primary sclerosing cholangitis (PSC) is a recognised risk factor for hilar cholangiocarcinoma (hCCA). In selected patients, neoadjuvant chemoradiotherapy followed by liver transplantation provides the optimal chance of long-term survival. However, for the patient described in the present case report, at the time of the patient s treatment, the UK did not have an approved transplant programme for cholangiocarcinoma, and access to liver transplantation was limited, often necessitating upfront surgical resection despite its complexity and limited curative potential. The present study describes the case of a 52-year-old male patient with PSC who was diagnosed with hCCA and underwent an extended right hepatectomy. After 26 months, progressive liver dysfunction due to PSC-related cirrhosis prompted liver transplantation, which was approved following a lengthy appeals process. Over the following years, the patient developed metastases in the bowel, lungs and abdominal wall, all of which were successfully managed with surgical resections. He remained disease-free for 8 years following his initial diagnosis before developing intrahepatic recurrence. The tumour was HER2-positive, and the compassionate use of zanidatamab was initiated following progression on standard therapies. At the time of the writing of the present case report, the patient remained alive 101 months following this initial diagnosis. On the whole, the present case report highlights the potential impact of tumour biology and multimodal treatment in PSC-associated hCCA. The prolonged survival of the patient despite delayed transplant and metastatic recurrence suggests that PSC-related hCCA may follow a more indolent course compared to *de novo* cases. Future efforts are required to focus on tumour profiling and stratified therapeutic approaches to better guide treatment in this complex disease.

## Introduction

Hilar cholangiocarcinoma (hCCA) accounts for 50-60% of all cholangiocarcinomas (1,2). *De novo* hCCA is an aggressive malignancy, with 5-year survival rates of up to 40% in selected series, particularly when negative lymph node status and clear resection margins are achieved (1).

Primary sclerosing cholangitis (PSC) is a well-recognised risk factor for hCCA, with an annual incidence estimated between 0.2 and 1.5% following the diagnosis of PSC (3,4). Outcomes for hCCA arising in the context of PSC appear to differ markedly from *de novo* cases. In selected patients undergoing resection, the 5-year survival rate for PSC-associated hCCA may reach 60%, compared to 30-40% in the *de novo* group (1,3,5).

Further improvements in long-term survival have been achieved through the implementation of transplant-based protocols, most notably the Mayo Clinic protocol, which combines neoadjuvant chemoradiotherapy followed by liver transplantation (4,6). Of note, 10-year survival rates approaching 70% have been reported in carefully selected patients (3). However, challenges remain, namely strict selection criteria, dropout due to disease progression, false-positive diagnoses with no tumour in the explant, and increased vascular complications post-transplant due to prior chemoradiation (4).

Despite these advances, the UK did not have an approved liver transplant programme for cholangiocarcinoma at the time of the treatment of the patient described in the present case report, limiting access to transplant-based pathways. Organ shortage further restricts curative options in this patient population, and upfront surgical resection remains the mainstay of treatment, albeit with key technical challenges. Regardless of treatment modality, recurrence rates remain high, affecting ~50% of cases (3,4).

The present study describes a rare case of long-term survival in a patient with PSC-associated hCCA initially treated with extended liver resection. Liver transplantation was performed 2 years thereafter, an unorthodox point in the disease course. Despite multiple episodes of metastatic recurrence under immunosuppression, each was successfully managed. The case described herein highlights the potential importance of tumour biology in determining long-term outcomes in PSC-associated hCCA.

## Case report

**Figure 1:**
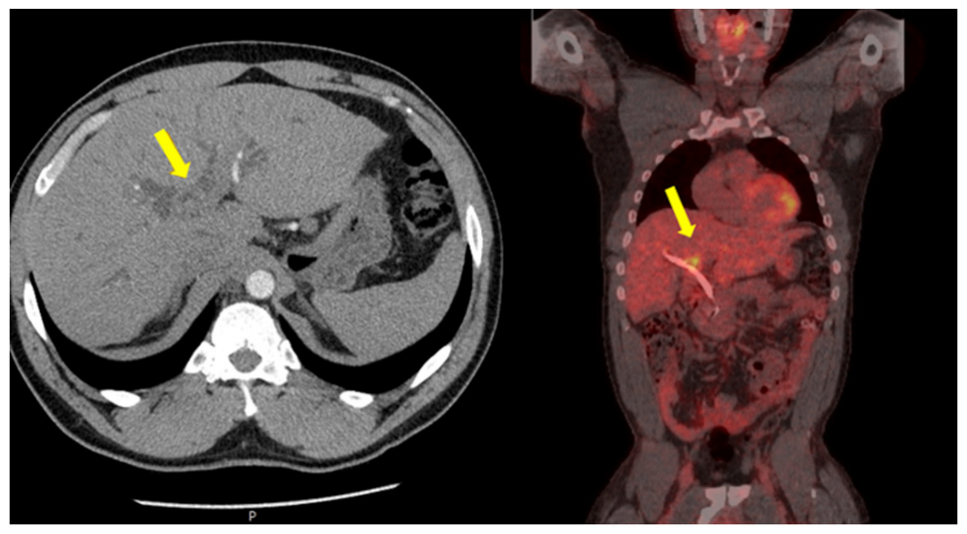
Axial computed tomography image (left panel) demonstrating bilateral intrahepatic biliary dilatation secondary to a hilar cholangiocarcinoma (yellow arrow). Coronal fluorodeoxyglucose positron emission tomography image (right panel) illustrating intense metabolic activity at the liver hilum corresponding to the hilar mass (yellow arrow). The image was obtained in May, 2017.

**Figure 2:**
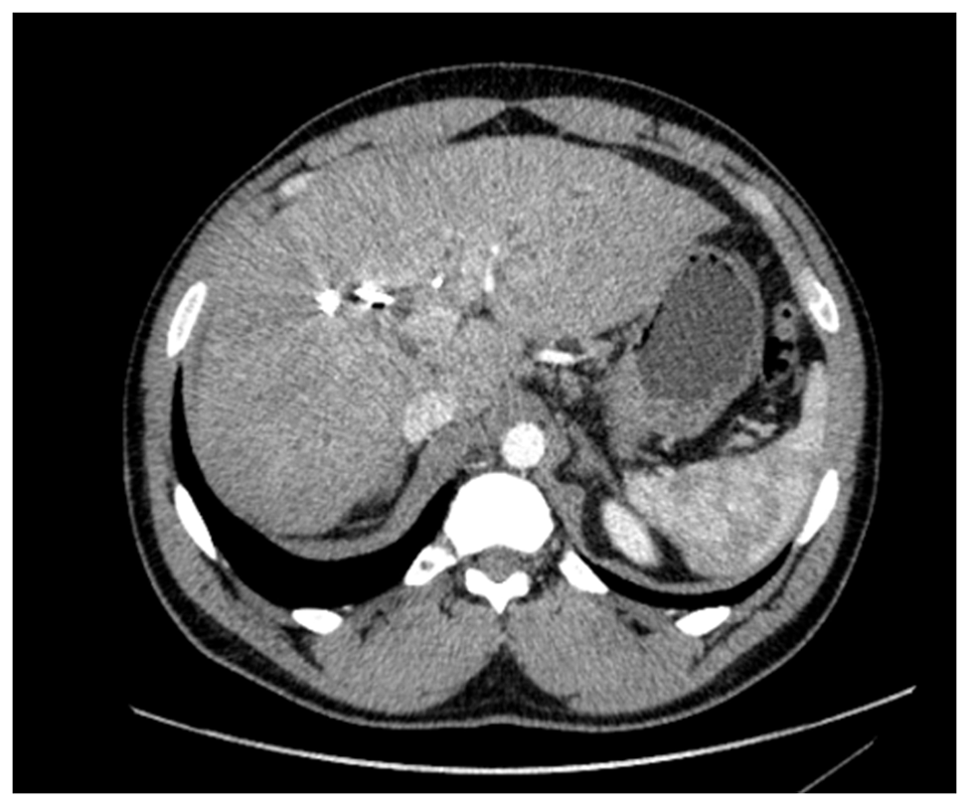
Axial computed tomography image demonstrating hypertrophy of the left lateral liver segment following portal vein embolization, performed in preparation for an extended right hepatectomy.

A 52-year-old male patient was diagnosed with PSC in 2015 at King s College Hospital (London, UK), confirmed by liver biopsy. He remained under regular surveillance by the hepatology team until April, 2017, when he presented with new-onset obstructive jaundice. Contrast-enhanced computed tomography (CT) revealed a hilar mass causing bilateral biliary dilation, and the working diagnosis was that of hCCA. After staging with fluorodeoxyglucose positron emission tomography (FDG-PET) and diagnostic laparoscopy, no evidence of metastatic disease was identified (Fig. 1). Portal vein embolization was performed to induce hypertrophy of the future liver remnant (FLR) in preparation for an extended right hepatectomy (ERH). Given the background of PSC, a 10-week interval was observed before repeat imaging, which demonstrated marginal FLR growth. The future liver remnant-to-body weight ratio (FLRBWR) was 0.42 at the time of surgery, corresponding to ~20% of total liver volume (Fig. 2).

**Figure 3:**
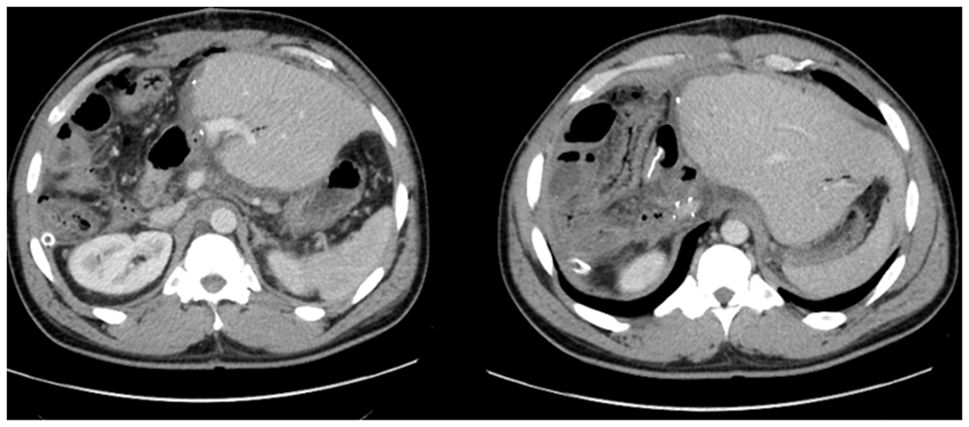
Axial computed tomography image illustrating the post-operative liver remnant following extended right hepatectomy.

In September 2017, the patient underwent ERH with Roux-en-Y hepaticojejunostomy and regional lymphadenectomy. Intraoperative portal pressure measurements exceeded 17 mmHg, prompting portal flow modulation with splenic artery ligation to mitigate the risk of post-hepatectomy liver failure (PHLF). The gastroduodenal artery was also ligated. The patient developed PHLF grade B, according to ISGLS criteria (7), although he did not meet the 50:50 criteria (8), and recovered well post-operatively. Pre-operative CA 19-9 was 43 U/ml (Fig. 3).

**Figure 4:**
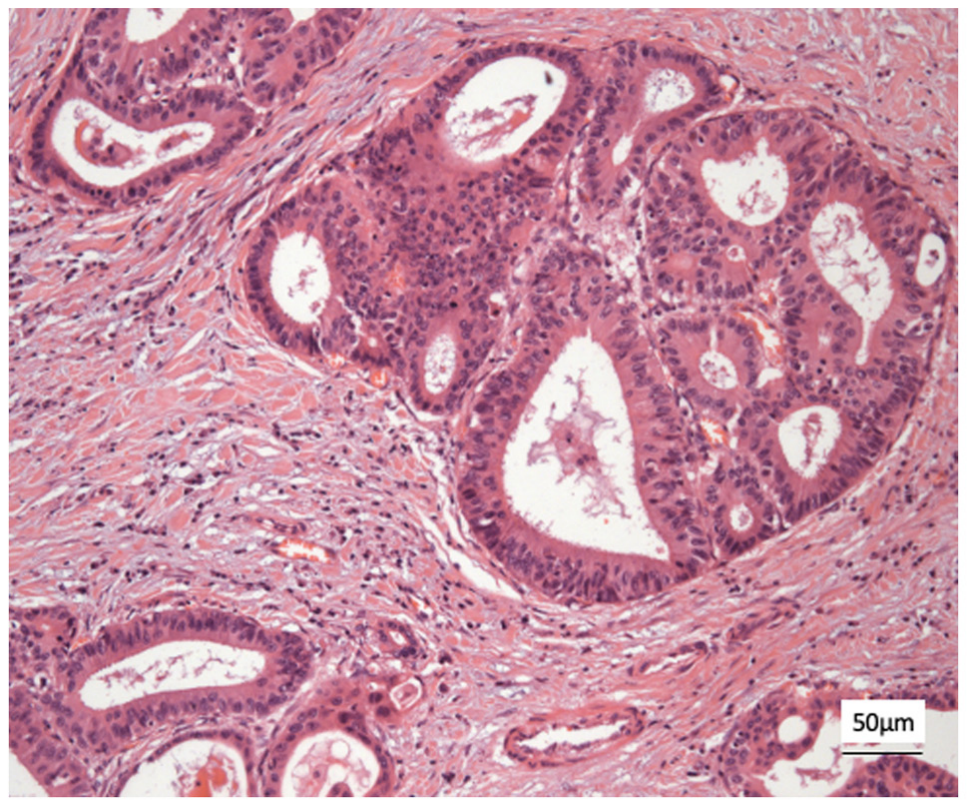
Haematoxylin and eosin staining demonstrating a moderately differentiated tubular adenocarcinoma, consistent with perihilar cholangiocarcinoma (original magnification, x400; indicative scale bar, ~50 µm).

Histological analysis revealed a moderately differentiated perihilar cholangiocarcinoma measuring 21 mm, arising in the setting of biliary intraductal papillary neoplasm of the extrahepatic ducts (30 mm) on a background of severe PSC. Proximal, distal, and circumferential margins were negative for malignancy. Perineural (PN) and lymphovascular (LV) invasion were both present. Final pathological staging was pT2aN0 (0/14) PN1LV1R0 (Fig. 4). Fig. 4 depicts routine haematoxylin and eosin (H&E) staining performed by the Department of Histopathology at King s College Hospital. Sections were paraffin-embedded and cut at a standard diagnostic thickness of 3-4 µm. Fixation was performed in 10% neutral-buffered formalin at room temperature for a minimum of 24 h. A standard H&E stain was used, procured from established hospital suppliers under ISO-accredited protocols. Staining was carried out using automated staining systems under standard diagnostic laboratory conditions. Microscopic evaluation was performed using a Leica DM1000 microscope. These procedures are in accordance with institutional diagnostic protocols.

Subsequently, the patient received two cycles of adjuvant capecitabine at 1,250 mg/m^2^ twice daily, administered on days 1-14 of a 21-day cycle. However, systemic therapy was discontinued in January, 2018 due to liver function deterioration. The patient remained disease-free on surveillance imaging for 18 months. He later developed progressive liver dysfunction and weight loss. Cross-sectional imaging revealed signs of progressive cholangiopathy and portal hypertension in addition to synthetic dysfunction. Given this, the patient underwent a successful appeal for listing and evaluation for liver transplantation.

**Figure 5:**
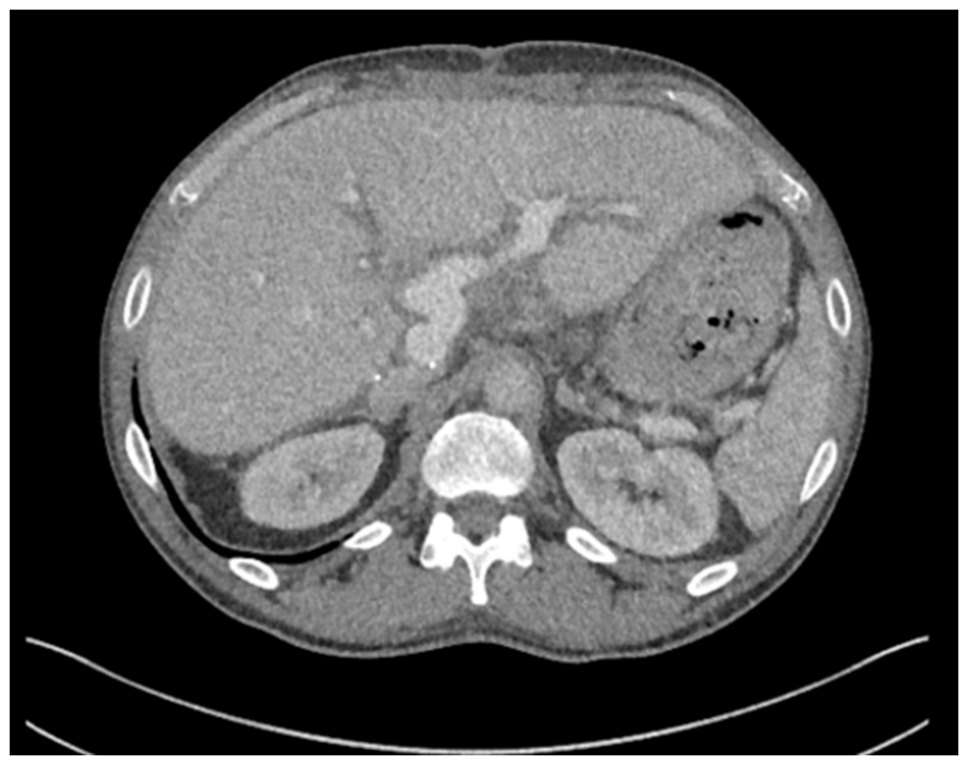
Orthotopic liver transplantation using a whole-liver graft from a brain-dead donor in 2019. The figure depicts a post-operative computed tomography scan performed in January, 2020, prior to discharge, after a long recovery from surgery.

In November, 2019, 26 months later, he underwent orthotopic liver transplantation using a whole liver graft from a brain-dead donor (cold ischemia time, 19 h) (Fig. 5). Intraoperatively, extensive adhesions involving the right colon resulted in a serosal tear requiring right hemicolectomy and temporary ileostomy.

A histopathological examination of the explanted liver revealed no evidence of residual hCCA. However, a 9-mm nodule on the serosal surface of the resected colon demonstrated histological features identical to the primary hCCA, confirming it as a metastatic deposit. This assessment was performed by the Department of Histopathology, King s College Hospital, using routine diagnostic protocols on formalin-fixed, paraffin-embedded tissue with standard haematoxylin and eosin (H&E) staining. Archived digital images of these slides are not available.

Following a prolonged recovery complicated by acute rejection, the patient was discharged in a stable condition. In February, 2020, following conversion to an mTOR-based immunosuppression regimen (9), he began treatment with adjuvant capecitabine at the standard BILCAP dose (1,250 mg/m^2^ twice daily, days 1-14 of a 21-day cycle) according to routine oncology protocols used for biliary tract cancers. However, treatment was interrupted after two cycles due to the COVID-19 pandemic. Surveillance imaging continued every 3 months.

**Figure 6:**
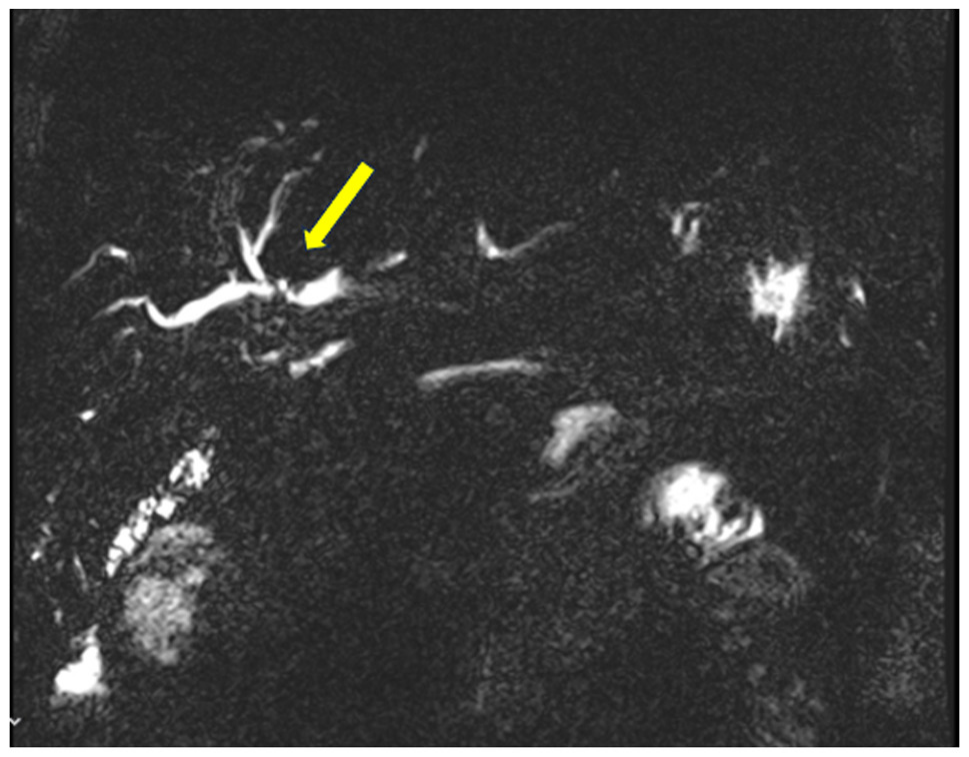
MRCP illustrating a new hilar stricture at the hepaticojejunostomy (yellow arrow), consistent with ischemic cholangiopathy in the liver graft (March, 2021). This was treated with balloon dilation.

In March 2021, a new hilar stricture was identified in the liver graft. Magnetic resonance cholangiopancreatography and biopsy revealed features of ischemic cholangiopathy, possibly from prior rejection, although recurrent PSC could not be excluded (Fig. 6). FDG-PET revealed no evidence of disease recurrence. A suspected anastomotic stricture at the hepaticojejunostomy was treated with balloon dilation via percutaneous transhepatic cholangiography, leading to the resolution of liver function abnormalities. The ileostomy was reversed in March, 2022.

**Figure 7:**
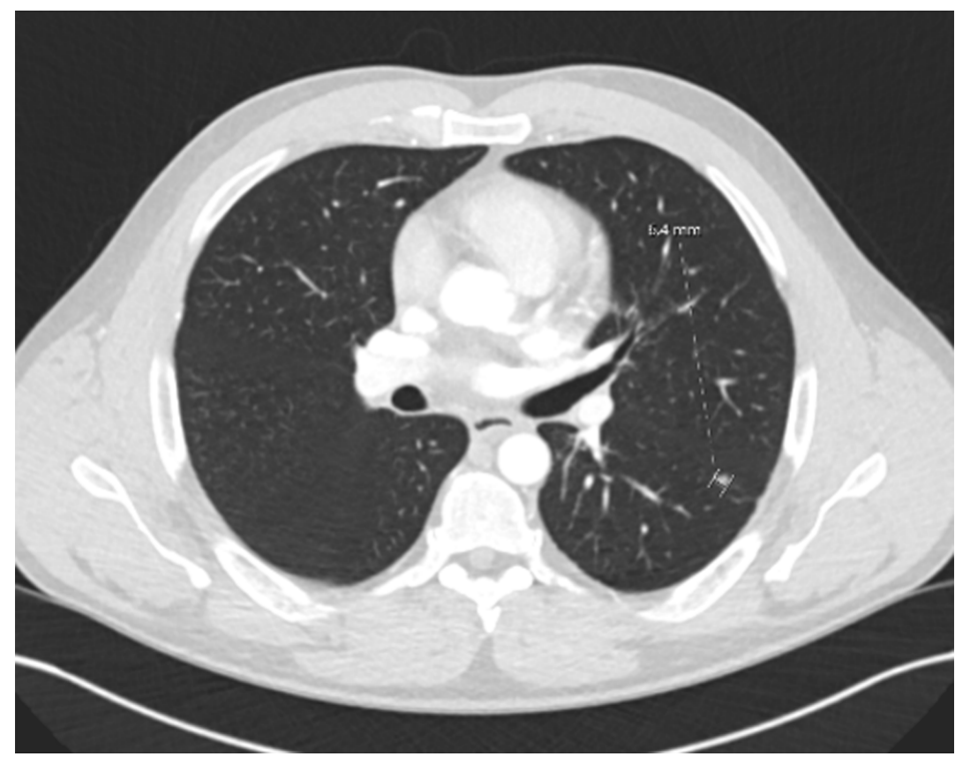
Axial contrast-enhanced computed tomography scan demonstrating a solitary pulmonary nodule in the left lower lobe, identified on routine surveillance in June, 2022.

The patient remained in a good condition clinically, although mild biochemical evidence of recurrent PSC was noted. In June, 2022, a solitary left lower lobe lung nodule was detected on surveillance imaging (Fig. 7). Wedge resection confirmed a 5-mm metastatic lesion from the original hCCA (pTisN0R0). The patient was commenced on capecitabine in November, 2022, typically administered at a dose of 1,250 mg/m^2^ twice daily on days 1-14 of a 21-day cycle. He completed seven cycles by April, 2023 with no observed toxicities.

**Figure 8:**
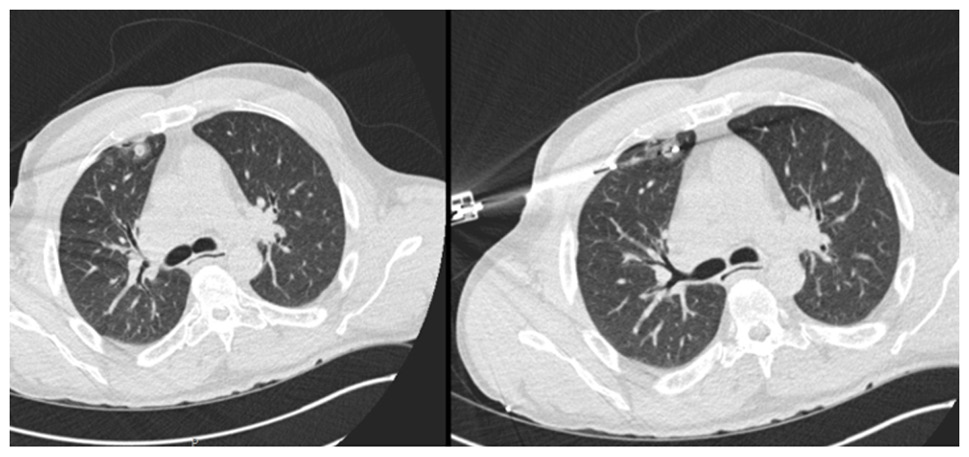
Axial computed tomography scan demonstrating a right upper lobe lung metastasis (left panel), subsequently treated with image-guided ablation (right panel).

**Figure 9:**
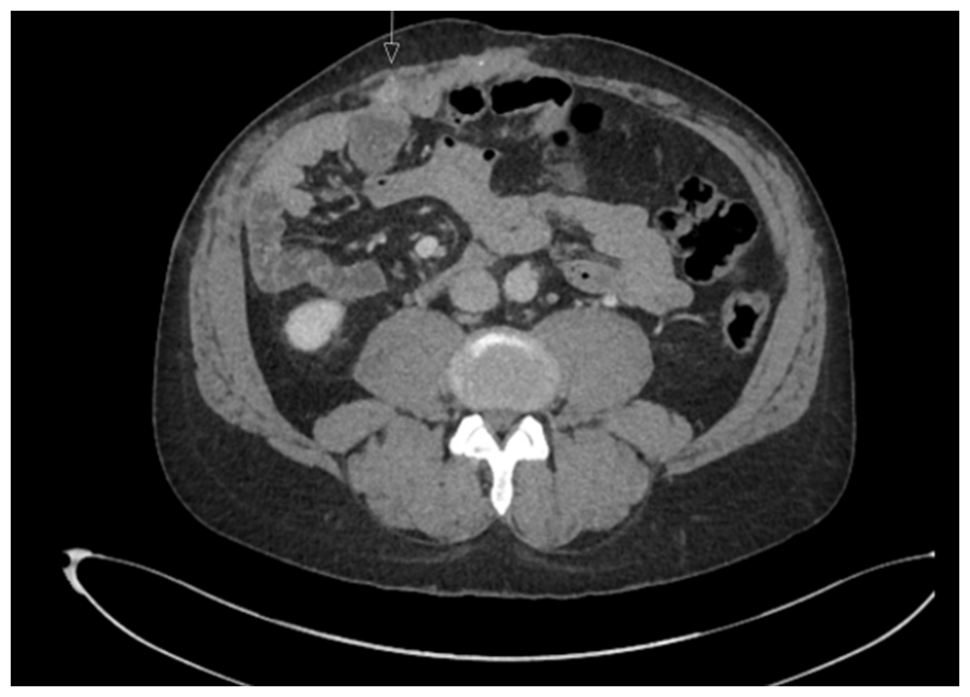
Axial computed tomography demonstrating an FDG-avid soft tissue lesion in the anterior rectus sheath, consistent with metastatic cholangiocarcinoma (white arrow).

In July 2023, PET imaging revealed a new FDG-avid lesion in the anterior rectus sheath. Diagnostic biopsy was performed with histology again confirmed metastatic hCCA. The patient began systemic palliative chemotherapy. The patient began systemic palliative chemotherapy with gemcitabine (1,000 mg/m^2^ on days 1 and 8) and cisplatin (25 mg/m^2^ on days 1 and 8 of each 21-day cycle). By January, 2024, following eight cycles, surveillance imaging demonstrated increased uptake in a previously stable lung nodule. The rectus sheath metastasis and lung lesion were treated with surgical excision and microwave ablation, respectively. A new solitary lung recurrence was treated with ablation in October, 2024 (Figs. 8 and 9).

**Figure 10:**
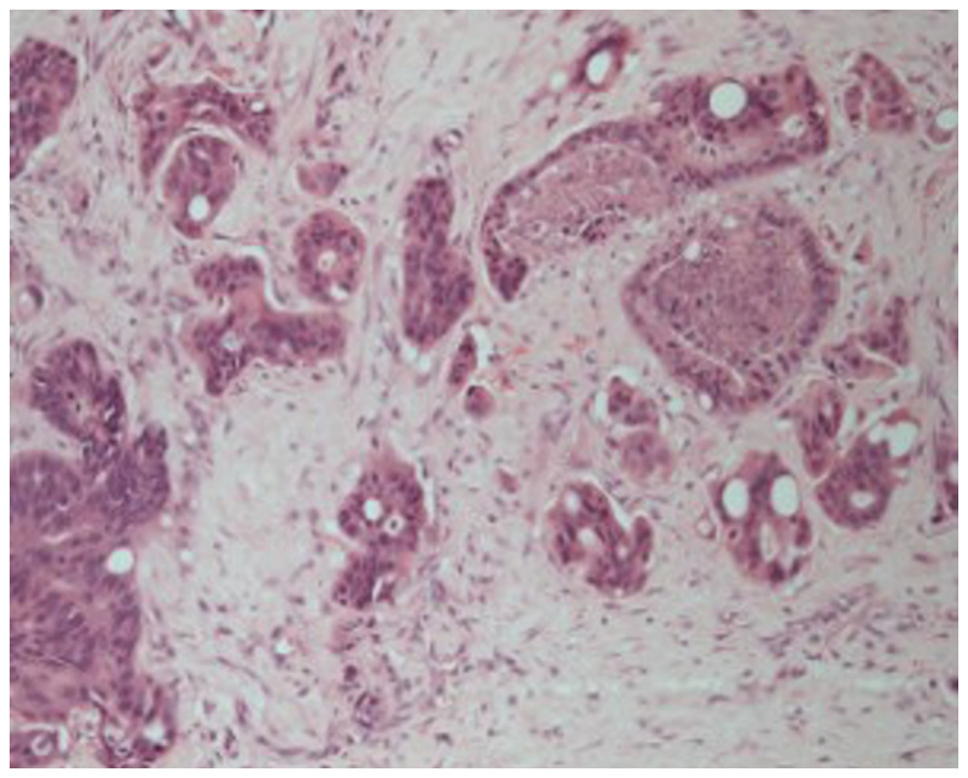
Haematoxylin and eosin staining of the rectus sheath metastasis, demonstrating moderately differentiated adenocarcinoma (magnification, x100; no calibrated scale bar available at source).

A histological examination of the rectus sheath deposit confirmed metastatic moderately differentiated adenocarcinoma, morphologically consistent with the patient s original diagnosis of hilar cholangiocarcinoma (Fig. 10). Histology was performed by the Department of Histopathology at King s College Hospital using routine paraffin-embedded sections, formalin fixation and standard diagnostic laboratory protocols.

**Figure 11:**
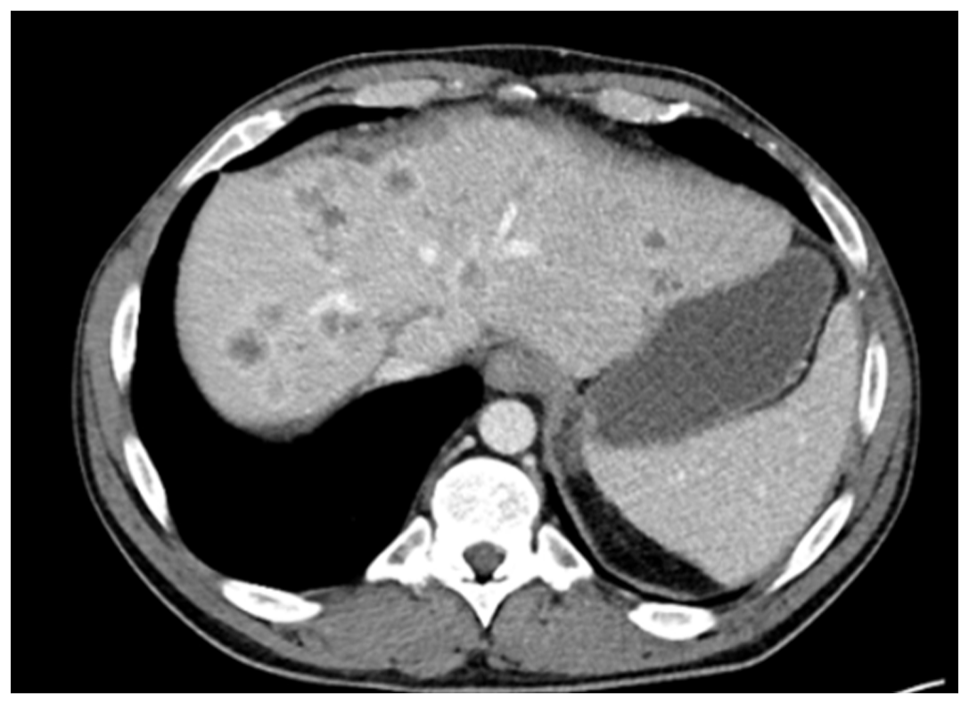
Axial computed tomography imaging illustrating multiple liver metastases in 2025, occurring 6 years following liver transplantation and 8 years following the initial diagnosis of PSC-related hilar cholangiocarcinoma.

In January, 2025, the patient developed multiple intrahepatic and peritoneal metastases (Fig. 11). Notably, HER2 overexpression was confirmed in the most recent metastatic specimen, prompting initiation of palliative systemic therapy with zanidatamab, a bispecific HER2-targeted antibody. HER2 immunohistochemistry was performed on 4-µm paraffin-embedded sections using the BOND ORACLE HER2 IHC System (cat. no. DS9800 Leica Biosystems), an automated, standardised assay in which all steps were executed according to the manufacturer s validated protocol.

**Figure 12:**
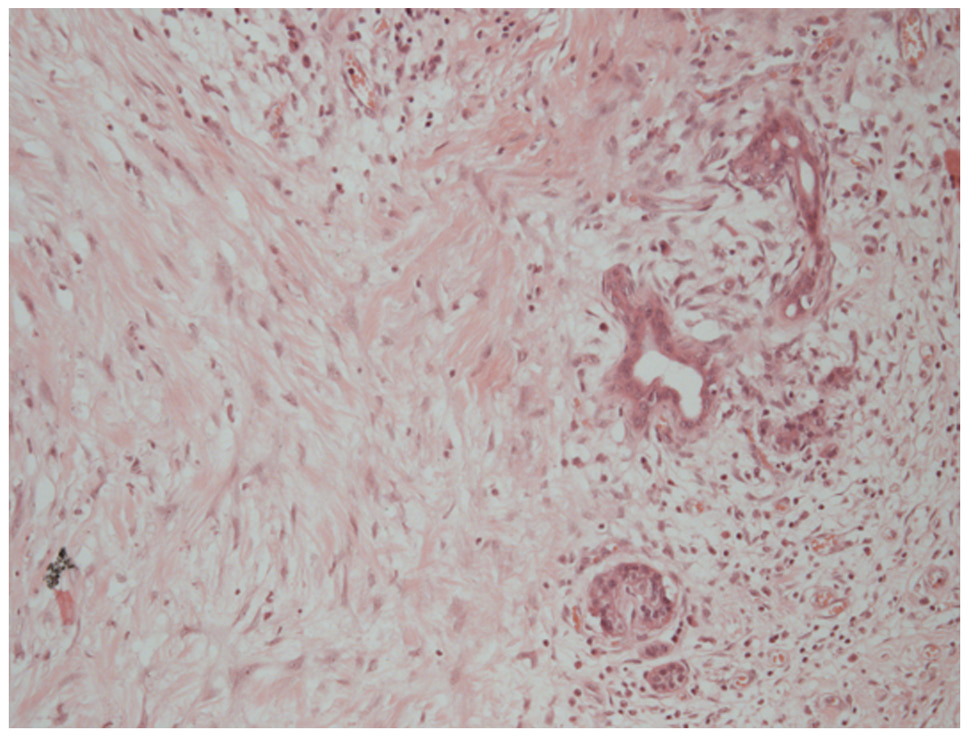
HER2 immunohistochemical staining illustrating membranous staining (score 2) (magnification, x200; no calibrated scale bar available at source). Slides were reviewed using a standard light microscope.

The primary hilar cholangiocarcinoma specimen resected in 2017 was retrospectively retrieved and subjected to the same HER2 IHC protocol, confirming HER2 overexpression in the original tumour (Fig. 12). Although HER2 testing was not routinely performed in 2017, the concordant HER2-positive status of both primary and metastatic sites supports the rationale for HER2-directed therapy in this patient.

Despite initial disease progression, he maintained a good performance status and received palliative chemotherapy, which was administered at a different institution. As such, detailed information regarding the specific agents and dosing schedule is unavailable. The patient ultimately passed away in November 2025, 103 months following his initial diagnosis of PSC-associated hilar cholangiocarcinoma in April, 2017. A summary of the disease course of the patient and key clinical events is provided in Table I.

## Discussion

hCCA is an aggressive malignancy often requiring extended liver resection to achieve oncological clearance and improve survival outcomes (1,10). The 5-year overall survival ranges between 30-40% (3,5), while the 5-year disease-free survival is reported to be ~20%, particularly when favourable features, such as well-differentiated tumours, negative lymph nodes and R0 margins are present (4,5,11).

PSC is a known predisposing factor, with an estimated lifetime risk of cholangiocarcinoma approaching 20%, and reported prevalence ranging from 6.5 to 13% (12). Accumulating evidence suggests that hCCA arising in the context of PSC has a more favourable prognosis compared to *de novo* hCCA. Patients with PSC-associated hCCA undergoing resection can achieve 5-year survival rates up to 60%, while the survival rates of those with *de novo* hCCA rarely exceed 30-40% (3,5).

This difference in outcomes is most notably demonstrated in transplant-based approaches pioneered by the Mayo Clinic, which developed the first structured protocol combining neoadjuvant chemoradiotherapy with liver transplantation for PSC-associated hCCA. In carefully selected patients, this strategy has achieved 10-year survival rates approaching 67% (3,4), significantly outperforming outcomes observed in *de novo* hCCA. However, its success is contingent upon stringent selection criteria, with dropout rates reaching 15% and up to 5% of explanted livers found to be tumour-free on final histology (4). Despite these limitations, the Mayo Protocol remains the gold standard and the most effective therapeutic framework for long-term survival in PSC-associated hCCA.

Recurrence remains a key concern. Even with liver transplantation, recurrence has been reported in up to 50% of cases (3,4), although this decreased to as low as 22% in carefully selected PSC hCCA cases treated as per the Mayo protocol (4).

The prospective study by Groot Koerkamp *et al* (13) in 2015 evaluated time to recurrence and disease-free survival in 306 patients with resected hCCA. The overall recurrence rate was 58%, with local recurrence in 26% and combined local and distant recurrence in 40% of cases (13). Isolated distant recurrence was not clearly documented, and only 2 patients experienced recurrence >8 years following resection. The median overall survival following recurrence was only 8 months (13). Notably, PSC-associated hCCA cases were not separately analysed (13).

The present study described the case of a patient with an isolated distant recurrence of PSC-associated hCCA occurring 5 years following initial resection and 2 years after liver transplantation, under immunosuppression. The serosal metastatic deposit discovered incidentally at the time of transplantation may represent the earliest sign of recurrence. However, there was no evidence of widespread progression during the subsequent 3 years of immunosuppression. Later recurrences in the lung and anterior abdominal wall remained solitary and were amenable to resection or ablation, with histology in some cases confirming only *in situ* disease. These features suggest a less aggressive biological phenotype, raising the possibility that tumour biology, rather than treatment modality alone, may play a key role in long-term survival.

Late-onset, isolated distant metastases are extremely rare. A similar case in the literature describes a solitary rib metastasis developing 10 years following the resection of *de novo* hCCA, treated with surgical excision (14). However, to the best of our knowledge, the present case report is the first to describe multiple late metastatic recurrences following an unorthodox treatment sequence, including delayed liver transplantation, with long-term survival >8 years.

A key point of discussion is the paradox between immunosuppression and tumour behaviour following transplantation. While chronic immunosuppression is generally associated with an increased oncological risk, emerging evidence suggests that immunosuppressive regimens containing mTOR inhibitors may have antitumour properties by inhibiting angiogenesis and tumour proliferation. In the patient in the present study, conversion to an mTOR-based immunosuppressive regimen shortly following liver transplantation may have contributed to delayed metastatic recurrence, synergising with the underlying favourable tumour biology (9).

Advances in biomarker discovery for PSC-related hCCA may support earlier diagnosis (15), while molecular profiling of cholangiocarcinoma is likely to enhance histological subtyping and facilitate the development of personalised treatment algorithms. In the case described herein, the tumour on the latest resected recurrence site was found to be HER2-positive, allowing the palliative initiation of zanidatamab, a bispecific HER2-targeted antibody recently shown to be effective in HER2-amplified biliary tract cancers (16).

Notably, the retrospective assessment of the primary tumour specimen resected in 2017 confirmed HER2 overexpression, corroborating the findings from the metastatic site and further supporting the rationale for HER2-directed therapy at this final stage.

HER2 overexpression has been identified in ~10-15% of biliary tract cancers (BTCs), including extrahepatic cholangiocarcinoma. Several studies have explored the prognostic relevance of HER2 overexpression in BTC, though findings have varied. In 2020, Vivaldi *et al* (17) reported HER2 overexpression and associated it with a significantly shorter disease-free survival, suggesting a more aggressive biological behaviour. In 2021, the multicentre study by Hori *et al* (18) found no significant impact of HER2 expression on survival outcomes across intrahepatic and extrahepatic cholangiocarcinomas, thus challenging its utility as a prognostic marker. Recently, in 2024, Kim *et al* (19) demonstrated that HER2 amplification was significantly associated with a reduced overall survival, reinforcing its potential role as a negative prognostic factor in BTC and a rationale for HER2-targeted therapies.

Notably, routine HER2 testing in cholangiocarcinoma was not widely implemented in clinical practice until ~2020, coinciding with early-phase trials exploring HER2 blockade in biliary tract cancers. Retrospective HER2 assessment in older cases, such as the patient in the present study (diagnosed in 2017), was not standard practice at the time.

Biological factors may have contributed to the relatively indolent clinical course observed in the patient described herein. PSC-associated cholangiocarcinoma often arises in a chronically inflamed and fibrotic biliary microenvironment, which promotes stepwise neoplastic transformation, but may also limit angiogenesis and metastatic dissemination (2,15). Compared with *de novo* cases, PSC-related tumours have been shown to exhibit lower proliferative indices, dense lymphocytic infiltration and a dominant inflammatory stroma, features that may contribute to a more immunologically active and less aggressive phenotype (4,15,20). Additionally, mismatch repair deficiency and microsatellite instability, observed in a subset of PSC-related cholangiocarcinomas, may underlie reduced tumour growth kinetics and more favourable treatment responses s(21).

In conclusion, organ shortage and limited transplant programme availability, as was the case in the UK at the time of the treatment of the patient described herien, continue to limit access to potentially curative strategies for patients with PSC-associated hCCA. In such settings, upfront resection remains the mainstay of treatment despite its technical demands and uncertain long-term efficacy.

The superior survival outcomes observed in PSC-associated hCCA, both with resection and transplant-based protocols, may reflect an underlying difference in tumour biology compared to de novo cases. The case described herein underscores the potential role of tumour biology as a key prognostic driver, particularly when durable survival is achieved despite recurrence and long-term immunosuppression.

Late isolated distant metastasis is exceptionally rare in hCCA. The present case report demonstrates that long-term survival and the control of metastatic disease may be achievable in highly selected patients through multimodal treatment and biologically favourable tumour behaviour.

## Figures and Tables

**Table I:** Timeline summary of the course of the disease.

1.	2015: Diagnosis of PSC
2.	April 2017: Diagnosis of hCCA
3.	September 2017: ERH with Roux enY hepaticojejunostomy
	Histology pT2N0PN1LV1R0
	SACT two cycles discontinued due to liver dysfunction
4.	November 2019: Liver transplantation and right hemicolectomy, terminal ileostomy
	Incidental finding of serosa deposit on right colon
	DBD whole graft CIT 19 h
5.	March 2022: GI restoration
6.	August 2022: Lung wedge resection for pTisN0R0 lung deposit
	SACT seven cycles of adjuvant capecitabine completed
7.	July 2023: New deposit on rectus sheath and new Lung lesion
	SACT eight cycles Aug 23-Jan 24 of palliative gemcitabine/cisplatin completed
	April 2024: Resection of rectus sheath deposit and ablation of lung metastasis
8.	October 2024: Another lung deposit treated with ablation
9.	January 2025: CT multiple liver metastases with peritoneal spread
	SACT commenced FOLFOX palliative chemotherapy (cycle 5 received April, 2025)
10.	August 2025: Disease progression on FOLFOX; commenced compassionate-use Zanidatamab for HER2-positive cholangiocarcinoma
11.	November 2025: Patient succumbed to the disease

PSC, primary sclerosing cholangitis; hCCA, hilar cholangiocarcinoma; ERH, extended right hepatectomy; SACT, systemic anti-cancer therapy; DBD, donation after brain death; CIT 19 h, 19-h cold ischemia time; GI, gastrointestinal; CT, computed tomography; FOLFOX, folinic acid, fluorouracil and oxaliplatin.

## Data Availability

The data generated in the present study may be requested from the corresponding author.

## References

[1] Soares KC, Jarnagin WR (2021). The landmark series: Hilar cholangiocarcinoma. Ann Surg Oncol.

[2] Banales JM, Marin JJG, Lamarca A, Rodrigues PM, Khan SA, Roberts LR, Cardinale V, Carpino G, Andersen JB, Braconi C (2020). Cholangiocarcinoma 2020: The next horizon in mechanisms and management. Nat Rev Gastroenterol Hepatol.

[3] Tan EK, Taner T, Heimbach JK, Gores GJ, Rosen CB (2020). Liver transplantation for peri-hilar cholangiocarcinoma. J Gastrointest Surg.

[4] Villard C, Jorns C, Bergquist A (2024). Treatment of cholangiocarcinoma in patients with primary sclerosing cholangitis: A comprehensive review. eGastroenterology.

[5] Kang MJ, Jang JY, Chang J, Shin YC, Lee D, Kim HB, Kim SW (2016). Actual long-term survival outcome of 403 consecutive patients with hilar cholangiocarcinoma. World J Surg.

[6] Heimbach JK, Gores GJ, Haddock MG, Alberts SR, Nyberg SL, Ishitani MB, Rosen CB (2004). Liver transplantation for unresectable perihilar cholangiocarcinoma. Semin Liver Dis.

[7] Rahbari NN, Garden OJ, Padbury R, Brooke-Smith M, Crawford M, Adam R, Koch M, Makuuchi M, Dematteo RP, Christophi C (2011). Posthepatectomy liver failure: A definition and grading by the international study group of liver surgery (ISGLS). Surgery.

[8] Balzan S, Belghiti J, Farges O, Ogata S, Sauvanet A, Delefosse D, Durand F (2005). The ‘50-50 criteria’ on postoperative day 5: An accurate predictor of liver failure and death after hepatectomy. Ann Surg.

[9] Semaan S, Connor AA, Saharia A, Kodali S, Elaileh A, Patel K, Soliman N, Basra T, Victor DW III, Simon CJ (2025). Transplantation for peri-hilar and intrahepatic cholangiocarcinoma with mTOR immunosuppression. Transplant Proc.

[10] Gilbert TM, Hackett J, Holt L, Bird N, Quinn M, Gordon-Weeks A, Diaz-Nieto R, Fenwick SW, Malik HZ, Jones RP (2022). Long-term morbidity after surgery for perihilar cholangiocarcinoma: A cohort study. Surg Oncol.

[11] Nooijen LE, Banales JM, de Boer MT, Braconi C, Folseraas T, Forner A, Holowko W, Hoogwater FJH, Klümpen HJ, Groot Koerkamp B (2022). Impact of positive lymph nodes and resection margin status on the overall survival of patients with resected perihilar cholangiocarcinoma: The ENSCCA registry. Cancers (Basel).

[12] Safarpour AR, Askari H, Ejtehadi F, Azarnezhad A, Raeis-Abdollahi E, Tajbakhsh A, Abazari MF, Tarkesh F, Shamsaeefar A, Niknam R (2021). Cholangiocarcinoma and liver transplantation: What we know so far?. World J Gastrointest Pathophysiol.

[13] Groot Koerkamp B, Wiggers JK, Allen PJ, Besselink MG, Blumgart LH, Busch OR, Coelen RJ, D Angelica MI, DeMatteo RP, Gouma DJ (2015). Recurrence rate and pattern of perihilar cholangiocarcinoma after curative intent resection. J Am Coll Surg.

[14] Ota Y, Matsuyama R, Taniguchi K, Ueda M, Takeda K, Tanaka K, Nakayama T, Endo I (2013). Solitary rib recurrence of hilar cholangiocarcinoma 10 years after resection: Report of a case. Clin J Gastroenterol.

[15] Catanzaro E, Gringeri E, Burra P, Gambato M (2023). Primary sclerosing cholangitis-associated cholangiocarcinoma: From pathogenesis to diagnostic and surveillance strategies. Cancers (Basel).

[16] Harding JJ, Fan J, Oh DY, Choi HJ, Kim JW, Chang HM, Bao L, Sun HC, Macarulla T, Xie F (2023). Zanidatamab for HER2-amplified, unresectable, locally advanced or metastatic biliary tract cancer (HERIZON-BTC-01): a multicentre, single-arm, phase 2b study. Lancet Oncol.

[17] Vivaldi C, Fornaro L, Ugolini C, Niccoli C, Musettini G, Pecora I, Cacciato Insilla A, Salani F, Pasquini G, Catanese S (2020). HER2 overexpression as a poor prognostic determinant in resected biliary tract cancer. Oncologist.

[18] Hori Y, Yoh T, Seo S, Minamiguchi S, Haga H, Taura K (2021). Limited Impact of HER2 Expression on Survival Outcomes in Patients with Intrahepatic Cholangiocarcinoma After Surgical Resection. Oncologist.

[19] Kim Y, Jee S, Kim H, Paik SS, Choi D, Yoo SH, Shin SJ (2024). EGFR, HER2, and MET gene amplification and protein expression profiles in biliary tract cancer and their prognostic significance. Oncologist.

[20] Oura K, Morishita A, Nakahara M, Tadokoro T, Fujita K, Tani J, Masaki T, Kobara H (2025). Chronic Liver Disease Associated Cholangiocarcinoma: Genomic Insights and Precision Therapeutic Strategies. Cancers (Basel).

[21] Goeppert B, Roessler S, Renner M, Singer S, Mehrabi A, Vogel MN, Pathil A, Czink E, Köhler B, Springfeld C (2019). Mismatch repair deficiency is a rare but putative therapeutically relevant finding in non-liver fluke associated cholangiocarcinoma. Br J Cancer.

